# Biological Deacidification and High-Value Transformation of Acidic Citrus Pulp by Multi-Microbial Fermentation

**DOI:** 10.3390/foods15081276

**Published:** 2026-04-08

**Authors:** Wei Xian, Xueling Qin, Xi Hu, Yusheng Liang, Hong Xie, Tao Pan, Zhenqiang Wu

**Affiliations:** 1 School of Biology and Biological Engineering, South China University of Technology, Guangzhou 510006, China; 2 Jiangxi Provincial Key Laboratory of Environmental Pollution Prevention and Control in Mining and Metallurgy, Jiangxi University of Science and Technology, Ganzhou 341000, China

**Keywords:** citrus pulp, biological deacidification, multi-microbial fermentation, metabolic pathway, biostimulant

## Abstract

Excessive acidity restricts the utilization of citrus pulp, a major by-product of the dried tangerine peel industry. To overcome this bottleneck, a functional microbial consortium (BsHpMrF) comprising *Bacillus subtilis* L4, *Hanseniaspora pseudoguilliermondii* B4, and *Monascus ruber* CGMCC 10910 was constructed for efficient biological deacidification. The consortium exhibited a synergistic effect, achieving an 88.23% reduction in total acidity and converting the acidic pulp into a neutral, bio-stabilized substrate. Untargeted metabolomics analysis revealed that this efficiency was driven by the concurrent activation of the TCA cycle and glyoxylate shunt for organic acid mineralization, coupled with membrane lipid remodeling (increased unsaturation) to enhance acid tolerance. Notably, the fermentation process functioned as a “metabolic factory”, significantly enriching the matrix with bioactive lipids (e.g., 10-HDA, nervonic acid) and indole-3-acetic acid (IAA, 414.28 mg/L). Application assays demonstrated that the fermentation products acted as a potent biostimulant for soybean sprouts, significantly promoting lateral roots and eliciting the accumulation of antioxidant phenolics and flavonoids. This study provides a sustainable “waste-to-treasure” strategy, valorizing acidic citrus pulp into a functional biostimulant for high-quality edible sprout production, thereby achieving a sustainable “waste-to-food” circular loop.

## 1. Introduction

In the Xinhui citrus industry, the disproportionate focus on high-value medicinal peel has led to the widespread discarding of the acidic, seed-heavy pulp, resulting in significant resource waste and environmental pressure [1,2]. Nevertheless, citrus pulp is rich in carbohydrates, organic acids, polyphenols, and dietary fiber, indicating considerable potential for further valorization [3]. Driven by carbon neutrality targets and the concept of circular agriculture, the sustainable utilization and high-value conversion of citrus processing by-products have become important priorities for the citrus industry [4]. Successful deacidification could unlock diverse application pathways for this by-product. Specifically, neutralized citrus pulp can be transformed into improved animal feed with enhanced palatability [5], as well as nutrient-rich biostimulants for sustainable horticulture [6].

Citric acid and malic acid are the predominant organic acids in citrus pulp and play decisive roles in sourness perception and sensory acceptance. Despite its nutritional value and application potential, the inherently high acidity of citrus pulp remains a critical bottleneck limiting its further processing and utilization. Conventional deacidification technologies in fruit and vegetable processing mainly include chemical neutralization, ion exchange, adsorption, and membrane-based separation. Although these approaches can reduce organic acid content and adjust the sugar–acid balance, they are often associated with drawbacks such as flavor deterioration, nutrient loss, and high operational costs [7,8].

In recent years, biological deacidification has attracted increasing attention due to its mild processing conditions, absence of chemical residues, and potential to improve sensory quality [9]. Various microorganisms have been reported to possess organic acid-degrading capabilities. Among them, lactic acid bacteria (LAB) are the most widely applied agents, primarily known for converting malic acid into lactic acid via malolactic fermentation [10]. However, the application of LAB in high-acid agricultural substrates is often limited. Their metabolic strategy typically involves an “acid-to-acid” conversion, where stronger organic acids are transformed into lactic acid, resulting in residual acidity and a limited increase in pH rather than complete neutralization [11,12]. Furthermore, high concentrations of citric acid in citrus residues can inhibit the growth of many LAB strains or divert their metabolism toward the production of undesirable metabolites such as acetic acid [13]. To overcome these limitations, non-lactic acid bacteria consortia offer a more promising strategy for thorough deacidification. *Bacillus subtilis* can promote substrate degradation through its diverse extracellular enzyme system and efficiently mineralize citric acid into carbon dioxide and water via the tricarboxylic acid (TCA) cycle, significantly elevating the pH [14]. Non-*Saccharomyces* yeasts, such as *Hanseniaspora* spp., are capable of utilizing citric acid as a carbon source while modulating flavor formation through ester biosynthesis [15]. In addition, filamentous fungi like *Monascus* spp. maintain high metabolic activity under low-pH stress and can produce varying secondary metabolites that facilitate the fermentation process [16]. Despite these individual potentials, most previous studies have focused on single-strain fermentations in juice or alcoholic systems [1], with limited attention to agricultural by-products such as citrus pulp. Moreover, the synergistic deacidification mechanisms of multi-microbial consortia and their associated metabolic regulation remain largely unexplored.

In this context, metabolomics—particularly untargeted LC–MS- or GC–MS-based metabolomics—has emerged as a powerful tool for elucidating metabolic pathway remodeling and identifying key metabolites in fermented foods. Nevertheless, its systematic application to investigate biological deacidification mechanisms in fruits and fruit by-products remains limited [17,18].

Therefore, the present study developed a synergistic multi-microbial fermentation strategy for the valorization of acidic citrus pulp, utilizing a specialized consortium of *Hanseniaspora pseudoguilliermondii* B4, *Bacillus subtilis* L4, and *Monascus ruber* CGMCC 10910. Beyond achieving effective biological deacidification, this bioprocess aimed to transform citrus waste into a high-value, bio-stabilized resource. By integrating fermentation optimization, enzymatic activity profiling, and untargeted metabolomics, the underlying mechanisms of organic acid biotransformation and metabolic pathway coordination during multi-microbial cooperative fermentation were systematically elucidated. Furthermore, the potential of the fermented product as a potent biostimulant was validated through sprout growth assays. These findings provide mechanistic insights into microbial cooperative deacidification and offer a sustainable, scalable biotechnological approach for the upcycling of agricultural by-products into functional bio-resources.

## 2. Materials and Methods

### 2.1. Materials and Chemicals

Citrus pulp was obtained from Xinhui mandarin oranges (*Citrus reticulata* Blanco) produced in Jiangmen, Guangdong Province, China. Fruits were peeled, deseeded, and homogenized using a high-speed blender (Foshan Ouke Electric Appliance Co., Ltd., Foshan, China) to partially remove free juice. The resulting pulp was stored at −20 °C until use.

*Bacillus subtilis* L4 (Bs), *Hanseniaspora pseudoguilliermondii* B4 (Hp), and *Monascus ruber* CGMCC 10910 (Mr) were preserved at the School of Biology and Biological Engineering, South China University of Technology (Guangzhou, China).

Phosphoric acid, methanol, gallic acid, and hesperidin were purchased from Sigma–Aldrich (St. Louis, MO, USA). Folin—Ciocalteu reagent and the amino acid assay kit were obtained from Shanghai Macklin Biochemical Co., Ltd. and Sangon Biotech Co., Ltd. (Shanghai, China), respectively. All other reagents were of analytical grade and supplied by Zhiyuan Chemical Reagent Co., Ltd. (Tianjin, China).

### 2.2. Solid-State Fermentation of Citrus Pulp

Prior to inoculation, Hp was cultured in sterile YPD medium at 30 °C and 180 rpm for 24 h, Bs in sterile LB medium at 37 °C and 180 rpm for 24 h, and Mr in modified YPD medium (glucose 20 g, fish peptone 10 g, yeast extract 3 g, KCl 0.5 g, KH_2_PO_4_ 4 g, FeSO_4_·7H_2_O 0.01 g per liter, natural pH) at 30 °C and 180 rpm for 48 h.

Solid-state fermentation was conducted in 250 mL Erlenmeyer flasks, each loaded with 25 g of the substrate mixture (citrus pulp and wheat bran at a 20:5 *w*/*w* ratio). The initial moisture content of the fresh citrus pulp was 89%, and the resulting substrate mixture exhibited an initial moisture content of approximately 78%, providing an optimal environment for microbial growth. No additional moisture adjustments were made during the fermentation period. Wheat bran was utilized as a structural bulking agent to enhance porosity for gas exchange and as a supplementary nitrogen source to optimize the C/N ratio for the microbial consortium [19,20]. The flasks were then pasteurized at 80 ± 5 °C for 15 min. For inoculation, the microbial suspensions were standardized to a cell density of approximately 10^8^ CFU/mL (OD_600_ ≈ 1.0). A total of 2.5 mL of this suspension was added to each flask (corresponding to a 10% *v*/*w* inoculum size), achieving an initial inoculum density of 10^7^ CFU/g of dry substrate. The fermentation was carried out at 30 °C for 5 days without further moisture adjustment, ensuring that metabolic changes were driven solely by the microbial consortium under standardized initial conditions. The inoculation proportion for different fermentation modes were set at 1:1 and 1:1:1. Then, single-factor experiments and an L9 (3^4^) orthogonal experimental design were conducted using total acid as the evaluation index. Each fermentation was repeated three times.

After fermentation, samples were centrifuged at 7827× *g* (10,000 rpm) and 4 °C for 10 min, and the supernatants were collected for subsequent analyses. Samples were collected every 24 h during fermentation. The unfermented citrus pulp–wheat bran mixture was used as the control (F0).

### 2.3. Analysis of Deacidification Indicators and Nutritional Compositions

pH was measured using a PB-10 pH meter (Sartorius, Göttingen, Germany). Total acid was determined by titration with 0.1 M NaOH and expressed as mmol/mL NaOH equivalents. Reducing sugars were quantified using the DNS method [12]., while total sugars were determined by the phenol–sulfuric acid method [12]. Soluble protein content was determined according to the method described by the Bradford [21]. Free amino acid was quantified using a ninhydrin-based colorimetric assay kit according to the manufacturer’s protocol, with cysteine as the standard [22]. Results were expressed as μmol/mL of fermentation supernatant.

### 2.4. Determination of Organic Acids

Fermentation broth (5 mL) was centrifuged 7827× *g* (10,000 rpm), 10 min, 4 °C), diluted with ultrapure water, and filtered through a 0.22 μm membrane prior to analysis. Organic acids were quantified by HPLC using an 5C18-PAQ (4.6 μm × 150 mm) column with isocratic elution (0.05 M phosphate buffer, pH 2.5 (adjusted by phosphoric acid): methanol = 97:3). The injection volume was 10 μL, column temperature 30 °C, the flow rate was 0.8 mL/min, and the detection wavelength was 210 nm. Concentrations were calculated using external standard calibration curves [23].

### 2.5. Determination of Total Phenolic and Total Flavonoid Contents

Total phenolic content (TPC) was determined using the Folin—Ciocalteu method with gallic acid as the standard [24]. Diluted fermentation supernatant (100 μL) was reacted with 50 μL Folin—Ciocalteu reagent for 5 min in the dark, followed by addition of 150 μL 20% (*w*/*v*) Na_2_CO_3_ and adjustment to 1 mL with distilled water. After incubation at room temperature for 30 min in the dark, absorbance was measured at 760 nm, and TPC was expressed as mg gallic acid equivalents GAE/mL.

Total flavonoid content (TFC) was measured using a diethylene glycol colorimetric method with hesperidin as the standard [24]. Diluted fermentation supernatant (100 μL) was mixed with 400 μL blank reagent, 500 μL diethylene glycol, and 10 μL 160 g/L NaOH, followed by incubation at 40 °C for 10 min. After cooling to room temperature, absorbance was measured at 420 nm, and results were expressed as mg hesperidin equivalents (HE) per mL.

### 2.6. Enzyme Activity Assays

#### 2.6.1. Filter Paper Cellulase (FPase), Carboxymethyl Cellulase (CMCase), and Pectinase Activity Assays

The filter paper cellulase (FPase), carboxymethyl cellulase (CMCase) activity and pectinase activity were determined according to the method described by [25]. One unit of FPase activity was defined as the release of 1 μmol glucose per minute under the assay conditions. One unit of CMCase activity was defined as the amount of enzyme releasing 1 μmol glucose per minute under the assay conditions. One unit of pectinase activity was defined as the release of 1 mg galacturonic acid per minute under the assay conditions.

#### 2.6.2. Protease Activity

##### Extraction of Crude Protease

The fermentation broth was extracted with phosphate buffer (pH 7.5) for neutral protease or sodium lactate buffer (pH 3.0) for acidic protease at a 1:10 (*v*/*v*) ratio, followed by shaking at 30 °C and 180 rpm for 30 min. After centrifugation 7827× *g* (1000 rpm), 15 min, 4 °C), the supernatant was collected as the crude enzyme extract.

##### Protease Activity Assay

Protease activity was assayed by incubating 1 mL of diluted crude enzyme with 1 mL of 1% (*w*/*v*) acidic or neutral casein at 40 °C for 10 min, followed by termination with 2 mL of 0.4 M trichloroacetic acid [26]. After filtration, 1 mL of the filtrate was mixed with 5 mL of 0.4 M Na_2_CO_3_ and 1 mL of Folin—Ciocalteu reagent, and absorbance was measured at 680 nm. Protease activity was calculated based on L-tyrosine release. One unit of protease activity was defined as the amount of enzyme required to release 1 μmol of L-tyrosine per minute under the specified assay conditions.

### 2.7. Non-Targeted Metabolomics Analysis

#### 2.7.1. Sample Preparation

An aliquot (100 μL) of each sample was extracted with 400 μL methanol/acetonitrile (1:1, *v*/*v*) containing isotope-labeled internal standards. After vortexing (30 s) and sonication in an ice-water bath (10 min), the mixture was incubated at −40 °C for 1 h to precipitate proteins. Samples were then centrifuged at 11,270× *g* (12,000 rpm) for 15 min at 4 °C, and the supernatants were collected for UPLC–MS/MS analysis.

#### 2.7.2. UPLC-MS/MS Analysis

Polar metabolites were analyzed using a UHPLC system (Vanquish, Thermo Fisher Scientific, Waltham, MA, USA) equipped with a Waters ACQUITY UPLC BEH Amide column (2.1 mm × 50 mm, 1.7 μm) coupled to an Orbitrap Exploris 120 mass spectrometer (Thermo Fisher Scientific). The mobile phase consisted of 25 mmol/L ammonium acetate and 25 mmol/L ammonium hydroxide in water (pH 9.75; A) and acetonitrile (B). The autosampler temperature was set at 4 °C, and the injection volume was 2 μL. MS/MS data were acquired in information-dependent acquisition (IDA) mode using Xcalibur software (v4.4). ESI conditions were as follows: sheath gas 50 Arb, auxiliary gas 15 Arb, capillary temperature 320 °C, spray voltage 3.8 kV (positive) or −3.4 kV (negative), full MS resolution 60,000, MS/MS resolution 15,000, and stepped normalized collision energy of 20/30/40.

### 2.8. Determination of Indole-3-Acetic Acid (IAA) Content

IAA production was quantified via the Salkowski colorimetric assay [27]. Briefly, 100 μL of supernatant was mixed with an equal volume of fresh Salkowski reagent (1 mL of 0.5 M FeCl_3_ in 50 mL of 35% HClO_4_). After 30 min of dark incubation at room temperature, absorbance was measured at 530 nm.

### 2.9. Antioxidant Activity Analysis

The DPPH and ABTS radical scavenging activities were determined following the method of [28] with minor modifications. Briefly, 50 μL of diluted sample was mixed with 400 μL of either 60 μM DPPH solution or ABTS working solution (prepared by reacting 0.7 mM ABTS with 2.45 mM potassium persulfate for 16 h and adjusting absorbance to 0.70 ± 0.02 at 734 nm). The mixtures were incubated in the dark at room temperature for 30 min (DPPH) or 10 min (ABTS), after which absorbances were measured at 515 nm and 734 nm, respectively. Trolox was used as the standard, and all results were expressed as mg Trolox equivalents (TE)/g DW.

### 2.10. Sprout Cultivation and Morphological Characterization

Surface-sterilized soybean seeds were germinated at 25 °C for 48 h. Uniform seedlings were transplanted into hydroponic boxes containing 200 mL of either sterile ultrapure water (negative control, CK), Hoagland solution diluted with ultrapure water to 800–3000× (*v*/*v*), or BsHpMrF fermentation products diluted with ultrapure water to 800–3000× (*v*/*v*). All treatments were prepared using the same dilution ratios to ensure a standardized comparison of their bio-stimulatory effects. After 48 h of dark culture, morphological traits (stem length, root area, lateral roots) were analyzed using Image J, and fresh/dry weights were recorded. Dried sprouts (40 °C, 48 h) were ground and ultrasonically extracted (0.4 g powder in 6 mL water, 50 °C, 30 min) for phytochemical assays, including the determination of total phenolic content (TPC), total flavonoid content (TFC), and DPPH/ABTS radical scavenging activities.

### 2.11. Statistic Analysis

All experiments were performed in triplicate. Data are presented as mean ± standard deviation and analyzed by one-way ANOVA using SPSS 26.0 (IBM Corp., New York, NY, USA), with statistical significance set at *p* < 0.05. For untargeted metabolomics, raw MS/MS data were converted to the mzXML format using ProteoWizard (V3.0.24054). Peak detection, alignment, and identification were performed using a customized R-based pipeline integrated with the BiotreeDB (V3.0). Metabolite identification was strictly validated based on accurate mass (mass tolerance < 5 ppm) and MS/MS fragment patterns (Score > 80). Principal component analysis (PCA) was applied to visualize sample clustering, while orthogonal partial least squares–discriminant analysis (OPLS-DA) was used to identify group discrimination and significantly altered metabolites. Model robustness was validated by 200 permutation tests. Differential metabolites were screened based on VIP > 1, *p* < 0.05 (Student’s *t*-test), and |log_2_FC| ≥ 1. Correlations between metabolites and total acid were visualized using Gephi (v0.10), and pathway annotation was performed using the KEGG database.

## 3. Results and Discussion

### 3.1. Microbial Combinations and Fermentation Factors of Acid Conversion

**Figure 1:**
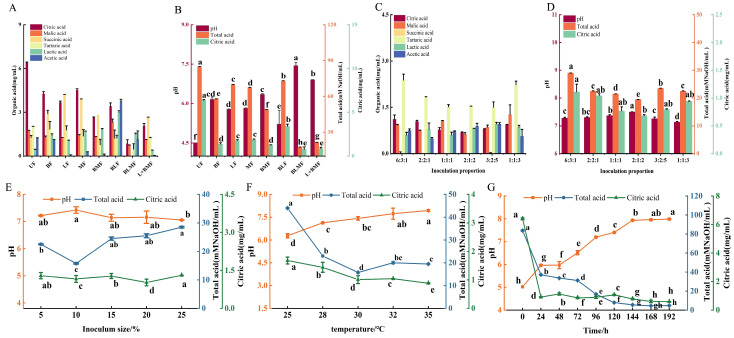
Screening of acid-reducing microbial consortia and optimization of fermentation parameters. (**A**) organic acids and (**B**) pH and total acid fermented by different microorganisms; (**C**) Changes in organic acids and (**D**) pH and total acid at different inoculation proportions; Effects of different fermentation conditions on the deacidification performance of the BsHpMrF consortium: (**E**) inoculum size (control inoculation proportion, temperature and time), (**F**) fermentation temperature (control inoculation proportion, inoculum size and time), (**G**) fermentation time (control inoculation proportion, inoculum size and temperature). Note: unfermentation (UF), Hp fermentation (HpF), Bs fermentation (BsF), Mr fermentation (MrF), various co-fermentations (HpMrF, HpBsF, BsHpMrF), inoculate Bs for 24 h and then inoculate Hp and Mr (Bs + HpMrF). Different letters (a, b, c, etc.) within the same time point indicate significant differences at *p* < 0.05 according to Duncan's multiple range test.

*Hanseniaspora pseudoguilliermondii* B4 was isolated and identified from lemon which could effectively degrade organic acids in citrus juice. However, its monoculture fermentation was insufficient to elevate the pH to neutrality [29]. To achieve efficient deacidification, *Monascus* spp., *Bacillus* spp., *Aspergillus niger*, and *Eurotium cristatum* capable of tolerating acidic environments and utilizing organic acids [16,30] were selected for co-fermentation with Hp, and the results are shown in Figure S1. The results indicated that co-fermentation of Hp with either Mr or Bs yielded a significantly superior deacidification effect compared to the Hp monoculture and other microbial combinations shown in Figure S1. Previous studies have confirmed that Mr as a crucial partner for deep deacidification [16], while Bs was exhibited significant effects in secreting hydrolytic enzymes and assisting oxidative acid mineralization [30]. Validation tests confirmed that the optimized consortium (BsHpMrF) outperformed all single-strain groups, elevating the pH from 4.51 ± 0.01 to 7.45 ± 0.02 (*p* < 0.05) (Figure 1). This superior efficacy demonstrates a strong synergistic interaction, justifying the selection of the BsHpMrF consortium for subsequent experiments.

Based on total acid as the primary evaluation index, the fermentation conditions of the selected microbial consortium were optimized for citrus pulp deacidification. Single-factor experiments showed that when the total inoculation amount was controlled at 10% and the temperature at 30 °C, fermentation for 120 h, the inoculation proportion of Bs: Hp: Mr was 2:1:2, the most effective deacidification was achieved, with the pH increasing to 7.49 ± 0.01 and total acid and citric acid contents decreasing to 19.5 ± 0.02 mM NaOH/mL and 0.68 ± 0.11 mg/mL, respectively. Meanwhile, the levels of the other five detected organic acids were also maintained at relatively low levels (Figure 1C).

Optimization of the individual fermentation parameters, with a fixed inoculum ratio (Bs: Hp: Mr) of 2:1:2, revealed that an inoculum size of 10% (*v*/*w*) and a temperature of 30 °C were most favorable for deacidification. Under these optimized conditions, the most pronounced acid-reducing effect was observed after 144 h of fermentation. The deacidification performance was significantly influenced by fermentation parameters. For inoculum size, the levels of total acid and citric acid initially decreased and reached their minimum at 10%, followed by a gradual increase at higher inoculation levels. For temperature and time, the acid concentrations significantly decreased before entering a stable plateau phase at 30 °C and 144 h, respectively. This suggested that an inoculum size of 10% and a temperature of 30 °C may provide the most favorable conditions for microbial growth, facilitating substrate decomposition and utilization to achieve optimal acid-reducing effects. During fermentation, pH continuously increased while total acid and citric acid decreased within 0~144 h, after which these parameters gradually stabilized. Based on these results, an L9 (3^4^) orthogonal experimental design was applied to further optimize key parameters. Range analysis revealed that the effects of the factors on total acid followed the order: time > inoculation size > temperature. Accordingly, the optimal fermentation conditions were determined as an inoculation proportion of 2:1:2, inoculation size of 10%, fermentation at 30 °C for 144 h (Table S2).

### 3.2. Mechanisms of Deacidification

#### 3.2.1. Organic Acids and Carbohydrates

**Figure 2:**
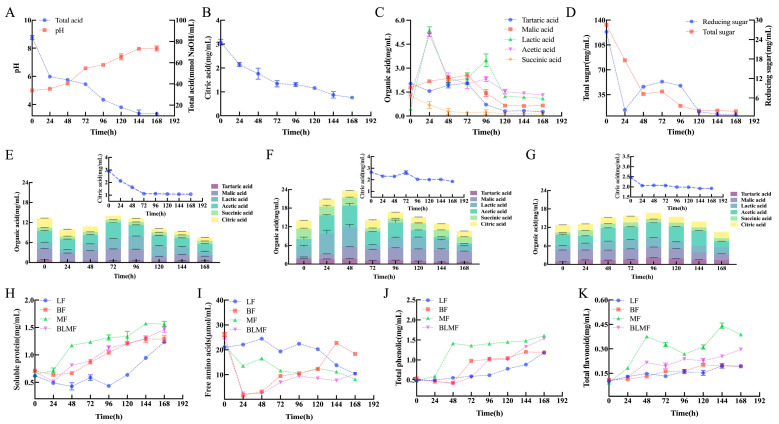
Fermentation-induced metabolic changes during microbial deacidification. (**A**) pH and total acid in BsHpMrF. (**B**) Citric acid content in BsHpMrF. (**C**) Selected organic acids in BsHpMrF. (**D**) Total and reducing sugar content in BsHpMrF. (**E**) Organic acids in Hp fermentation, the inset line chart (top right) shows the changes in citric acid content during HpF. (**F**) Organic acids in Bs fermentation, the inset line chart (top right) shows the changes in citric acid content during BsF. (**G**) Organic acids in Mr fermentation, the inset line chart (top right) shows the changes in citric acid content during MrF. (**H**) Soluble protein content. (**I**) Free amino acid content. (**J**) Total phenolic content. (**K**) Total flavonoid content. Note: Hp fermentation (HpF), Bs fermentation (BsF), Mr fermentation (MrF), multi-microbial fermentation (BsHpMrF).

Distinct organic acid dynamics clearly distinguished multi-microbial fermentation from single-strain fermentation during citrus pulp deacidification. For multi-microbial fermentation, total acid continuously decreased from 83.5 ± 0.06 to 12.25 ± 0.02 mmol NaOH/mL by 144 h, accompanied by a marked pH increase from 5.01 ± 0.03 to 7.90 ± 0.01 (Figure 2). Citric acid, the predominant organic acid, showed a sustained decline from 3.08 ± 0.07 to 0.87 ± 0.05 mg/mL, indicating efficient and continuous organic acid consumption. In contrast, single-strain fermentation exhibited clear limitations: Hp degraded only 60% of citric acid and showed transient accumulation of lactic and acetic acids; Bs fermentation caused a pronounced increase in lactic acid, leading to an initial rise in total acid; while Mr displayed slower organic acid turnover and lower citric acid degradation efficiency. These results demonstrated that individual microorganisms were insufficient for stable deacidification, whereas functional complementarity in the multi-microbial fermentation effectively prevented intermediate acid accumulation. Consistently, the levels of total sugar and reducing sugar in the mixed fermentation decreased significantly from their initial concentrations (133.05 ± 0.12 mg/mL and 26.33 ± 0.07 mg/mL, respectively) to 12.48 ± 0.11 mg/mL and 1.08 ± 0.02 mg/mL at the early stages of the process, indicating efficient carbon utilization that supported sustained organic acid metabolism. This synergistic effect was consistent with previous reports showing that Bs enhanced sugar release via hydrolytic enzymes and promoted TCA-associated acid metabolism [31], Bs directly utilized citric acid [32], and *Monascus* sp. maintained metabolic activity under low-pH conditions through secondary metabolism [33]. Collectively, rapid sugar consumption and continuous organic acid conversion acted cooperatively to drive efficient acidity reduction in citrus pulp.

#### 3.2.2. Chemical Composition

As shown in Figure 2H–K, the mixed fermentation group (BsHpMrF) exhibited more pronounced nutrient accumulation during the late fermentation stage than the single-strain groups. Specifically, soluble protein content in the BsHpMrF group reached approximately 1.32 ± 0.12 mg/mL at 144 h (Figure 2H). Although protein levels slightly increased until 168 h, 144 h was designated as the optimal endpoint as deacidification (Figure 2A) and bioactive compound accumulation (Figure 2J–K) had already reached a stable plateau, maximizing process efficiency. This effect was likely associated with extracellular proteases secreted by *B. subtilis*, which facilitate macromolecular protein degradation, while *H. pseudoguilliermondii* and *Monascus* sp. further assimilate the released nitrogenous compounds, resulting in positive metabolic synergy [15,34].

Variations in free amino acid content further revealed the dynamic nature of nitrogen metabolism within the consortium (Figure 2I). In the BsHpMrF group, free amino acid rapidly decreased to 2.48 ± 0.04 μmol/mL during early fermentation, followed by a gradual increase to 7.65 ± 0.07 μmol/mL at 144 h, suggesting initial assimilation for microbial growth and subsequent replenishment driven by sustained protein hydrolysis. In contrast, single-strain fermentations exhibited relatively limited fluctuations, indicating that microbial interactions markedly enhanced nitrogen turnover and metabolic flexibility. Previous studies have shown that maintaining amino acid homeostasis during fermentation supports microbial metabolic stability, influences organic acid consumption and flavor development through coupling with carbon metabolism [35].

Moreover, multi-microbial fermentation significantly promoted the accumulation of phenolic compounds (Figure 2J). Although some single-strain groups showed rapid initial increases, the total phenolic content (TPC) and total flavonoid content (TFC) in the BsHpMrF group (1.34 ± 0.01 mg/mL and 0.26 ± 0.03 mg/mL, respectively) demonstrated superior stability, more consistent levels at the optimized endpoint of 144 h compared to most monocultures. Although the levels of polyphenols and flavonoids in MrF were slightly higher than those in BsHpMrF, BsHpMrF achieved more thorough substrate utilization and better deacidification performance. This enhancement might result from hydrolytic enzymes produced by *B. subtilis* and yeast, which cleave cell wall polysaccharides and phenolic glycosides, together with secondary metabolites and environmental modulation provided by *Monascus* sp., favoring phenolic stabilization [33,36]. Similar synergistic effects have been widely reported in plant-based fermentations, where multi-strain systems generally outperform monocultures in enriching phenolic and flavonoid compounds [37,38].

### 3.3. Enzymolysis

**Figure 3:**
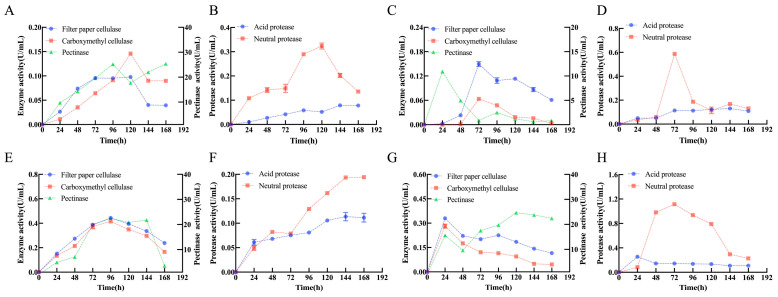
Changes in hydrolytic and protease activities among different fermentation groups during fermentation. (**A**) Hydrolytic activities in BsHpMrF, (**B**) Protease activities in BsHpMrF, (**C**) Hydrolytic activities in HpF, (**D**) Protease activities in HpF, (**E**) Hydrolytic activities in BsF, (**F**) Protease activities in BsF, (**G**) Hydrolytic activities in MrF, (**H**) Protease activities in MrF. Note: Hp fermentation (HpF), Bs fermentation (BsF), Mr fermentation (MrF), multi-microbial fermentation (BsHpMrF).

From an enzymatic perspective, functional differentiation among microorganisms within the multi-microbial system provided mechanistic insight into the enhanced deacidification performance. Overall, the multi-microbial fermentation (BsHpMrF) exhibited higher and more sustained activities of cellulolytic, pectinolytic, and proteolytic enzymes than any single-strain system. As fermentation time extended, the activities of cellulase, filter paper cellulase, and neutral protease initially increased and then decreased, while the activities of pectinase and acid protease exhibited an initial increase, followed by a decrease, and subsequently increased again. Specifically, filter paper cellulase, carboxymethyl cellulase, and pectinase activities in BsHpMrF increased markedly with fermentation time and reached elevated levels between 72 and 120 h. Filter paper cellulase peaked at approximately 0.10 ± 0.01 U/mL at 96 h. While the residual enzyme titers in the BsHpMr consortium were lower than the maximum observed in the *B. subtilis* monoculture (BsF, Figure 3E), the consortium maintained high metabolic efficiency. For instance, pectinase activity remained stable (17.72 ± 0.12 to 25.64 ± 0.10 U/mL) after 120 h, effectively supporting sustained substrate biotransformation (Figure 3A,C,E,G).

These results indicated that Bs acted as the primary contributor to cellulose and hemicellulose hydrolysis in the consortium, supplying fermentable sugars through its extracellular enzyme [39]. Protease activity further highlighted microbial complementarity. In the BsHpMrF group, neutral protease activity reached 0.32 ± 0.02 U/mL at 120 h (Figure 3B). While this residual activity was lower than the peak observed in the MrF monoculture (Figure 3G), it supported a more efficient metabolic flux. This indicates a synergistic consumption pattern where enzymes are rapidly utilized by the consortium to facilitate the biotransformation of citrus components, whereas in monocultures like MrF, enzymes tend to accumulate in the medium due to limited inter-species metabolic coupling [40]. While acidic protease activity increased steadily and remained at 0.08 ± 0.07~0.10 ± 0.05 U/mL during late fermentation. Although Mr exhibited high short-term neutral protease activity when cultured alone (up to 1.12 ± 0.12 U/mL at 48~72 h), this activity declined rapidly thereafter, suggesting a preference for transient intensive protein hydrolysis [33]. It is worth noting that the contents of organic acids, total sugar, and reducing sugar were also significantly consumed by 120 h, after which they tended to stabilize. This indicates that during fermentation, the hydrolytic enzymes and proteases secreted by the three strains achieved an effective synergistic effect, jointly promoting the utilization and conversion of organic acids and sugars. In addition, compared with single-strain fermentation, the mixed-culture fermentation prolonged the time required for hydrolases and proteases to reach their maximum activities, suggesting that mixed-culture fermentation may be more conducive to microbial decomposition and utilization of the substrate.

**Figure 4:**
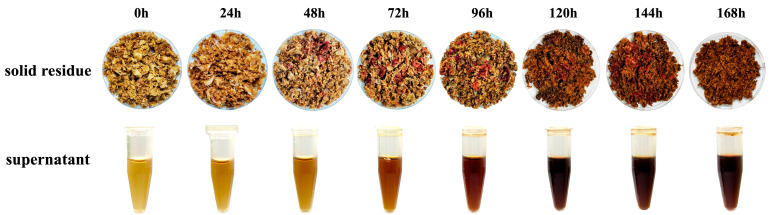
Visual appearance of solid residue and corresponding fermentation supernatants at different fermentation times.

Collectively, enhanced polysaccharide- and protein-degrading enzyme activities in BsHpMrF accelerated the release of reducing sugars and amino nitrogen, providing metabolic energy and precursors for organic acid conversion. This enzymatic synergy was consistent with the higher rates of total sugar and reducing sugar consumption observed in BsHpMrF and supported the efficient utilization of citrate and related organic acids via central carbon metabolism by Hp and Bs [9,32]. Meanwhile, protease production and secondary metabolism by Mr likely contributed to metabolic stability and interspecies coordination. Overall, the enhanced deacidification achieved by the consortium arises not from simple enzyme accumulation but from coordinated carbon–nitrogen metabolism driven by Bs-dominated polysaccharide hydrolysis, Hp-mediated organic acid regulation, and Mr -associated proteolysis and secondary metabolism. During the fermentation process, the fermentation substrate changed significantly (Figure 4). The pigments produced by Mr gradually deepen the red color of the fermentation substrate and its extract. Concurrently, active enzyme systems degraded cellulose and pectin in the substrate, resulting in a progressively finer and more viscous texture.

### 3.4. Metabolomic Model

To elucidate metabolic shifts during deacidification, UPLC–MS/MS-based untargeted metabolomics was conducted on samples from day 0 (F0) and day 6 (F6). Multivariate statistical analyses, including Principal component analysis (PCA) (Figure S2B) and orthogonal partial least squares discriminant analysis (OPLS-DA) (Figure S2C), revealed distinct metabolic separation between F0 and F6 with high intragroup consistency. The OPLS-DA model exhibited robust predictive performance (R^2^X = 0.81, R^2^Y = 1.00, Q^2^ = 0.998), and permutation testing (R^2^Y = 0.98, Q^2^ = −0.39; Figure S2D) confirmed the absence of overfitting. These results demonstrate the high reliability and stability of the metabolic profiles for subsequent differential metabolite identification.

#### 3.4.1. Differential Metabolites

**Figure 5:**
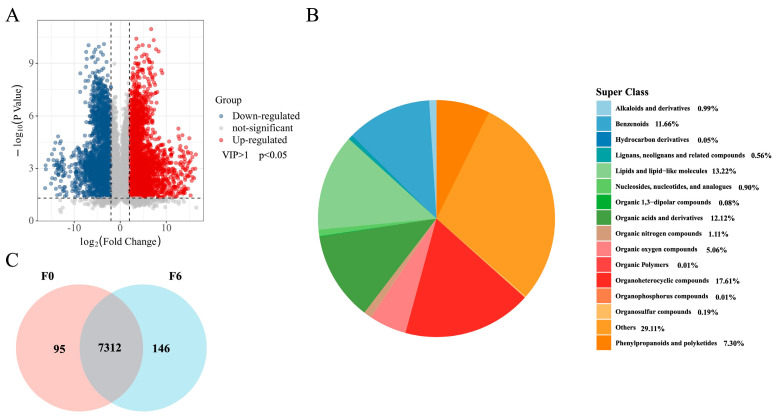
Distribution of metabolites in unfermented citrus pulp (F0) and fermented citrus pulp (F6). (**A**) Volcano plot showing the relationship between fold change and VIP values for metabolites comparing F0 and F6. (**B**) Pie chart illustrating the classification of the overall metabolites. (**C**) Venn diagram depicting the shared and unique differential metabolites between the F0 and F6 groups.

A total of 7547 metabolites were identified, with 5625 showing significant differential abundance (VIP > 1, *p* < 0.05) between F0 and F6 (Figure 5A). Among these, 2853 metabolites were upregulated, predominantly comprising lipids, organic acids, and phenylpropanoids, indicating extensive metabolic reprogramming. Specifically, pronounced accumulation was observed in lipid-like molecules (e.g., palmitoleic and pentadecanoic acids) and aromatic derivatives (Table S3), reflecting a systematic shift in substrate utilization. Venn analysis confirmed that 7312 metabolites were shared across groups (Figure 5C), further demonstrating that multi-microbial fermentation induced a global metabolic remodeling rather than isolated chemical changes.

#### 3.4.2. Correlation of Metabolites and Acids

**Figure 6:**
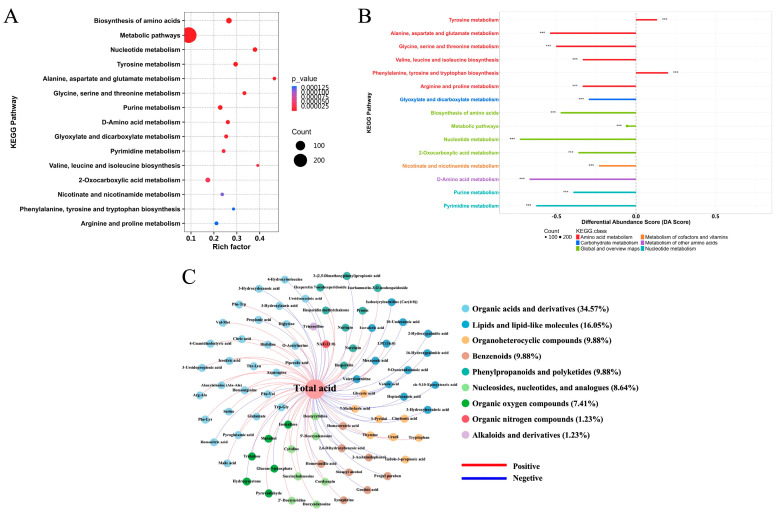
KEGG pathway enrichment analysis and correlation analysis between differential metabolites and total acid. (**A**) Bubble plot of KEGG pathway enrichment; (**B**) Differential abundance score plot of enriched KEGG pathways, where color indicates the *p* value of enrichment (darker colors represent lower *p* values and higher enrichment significance); *** indicate a highly significant difference at *p* < 0.001.(**C**) Correlation analysis between significantly altered metabolites and total acid. Node size reflects connectivity degree, edge color indicates correlation direction (red, positive; blue, negative), and edge thickness represents the absolute value of the correlation coefficient.

KEGG enrichment analysis revealed that differential metabolites were primarily associated with amino acid, nucleotide, and carbohydrate metabolism (Figure 6A). Most identified pathways, including the biosynthesis of branched-chain and aromatic amino acids, exhibited negative differential abundance scores (Figure 6B). This indicated a pronounced redistribution of carbon and nitrogen sources, likely fueled by the continuous consumption of organic acids [9].

Spearman correlation analysis identified 80 metabolites (|r| > 0.8, *p* < 0.05) closely linked to total acid degradation (Figure 6C). Among the 46 positively correlated metabolites, organic acids and their derivatives (e.g., malic acid, citric acid, and asparagine) showed sharp decreases (>98.5%), directly mirroring the pH increase and confirming organic acid metabolism as the central deacidification event. This might be attributed to the high-affinity transporters of yeast, which endow it with a strong capability to degrade organic acids [41]. Conversely, 34 metabolites were negatively correlated with total acid, dominated by lipids (32.35%) and benzenoids (20.50%). Significant accumulation was observed in specific lipid derivatives (e.g., pentanoylcarnitine and undecenoic acid) and aromatic compounds (e.g., indole-3-propionic acid), suggesting that multi-microbial fermentation channeled organic acid-derived carbon into lipid metabolism and aromatic transformation pathways [32,42]. The reduction in organic acids is often negatively correlated with lipid-related substances, the finding is consistent with previous study. The formation of lipid derivatives has also been reported to be associated with yeast metabolism, the enzymatic and chemical esterification of alcohols and acids during fermentation is significantly influenced by yeast strain [43].

### 3.5. Metabolic Pathways of Organic Acids

#### 3.5.1. Organic Acid Transformation

**Figure 7:**
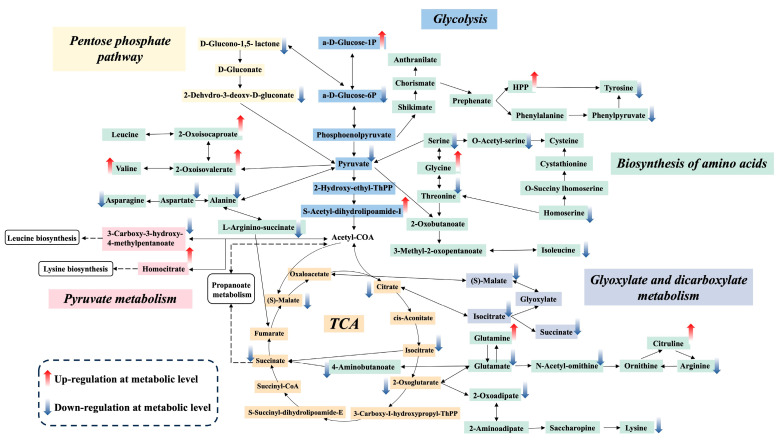
Metabolic pathway of organic acid transformation. Red and blue arrows indicate up-regulation and down-regulation based on MS-based untargeted metabolomic data (VIP > 1.0, *p* < 0.05). Pathway connections are based on the KEGG database.

During multi-microbial fermentation of citrus pulp, dynamic changes in metabolite profiles reflected the metabolic activities of different microorganisms at distinct fermentation stages and their cooperative interactions. Based on KEGG metabolic pathway enrichment results, the potential metabolic pathway of organic acid transformation was mapped (Figure 7). Untargeted metabolomic analysis revealed a marked decrease in α-D-glucose-6-phosphate (α-D-Glucose-6P) suggested sustained activation of glycolysis during fermentation. On one hand, α-D-Glucose-6P can be isomerized to β-D-glucose-6-phosphate and enter the pentose phosphate pathway, where D-gluconate is further converted into pyruvate via 2-dehydro-3-deoxy-D-gluconate and subsequently reintroduced into glycolysis. On the other hand, pyruvate generated through glycolysis is converted into acetyl-CoA via the intermediate S-acetyl-dihydrolipoamide-E, thereby entering the tricarboxylic acid (TCA) cycle. These metabolic features were consistent with the carbon flux redistribution and organic acid utilization characteristics of Hp, which has been shown to efficiently channel sugars and organic acids into central energy metabolism [9,32].

After 6 days of fermentation, the relative abundance of citrate decreased markedly, indicating its continuous consumption and further metabolic conversion. A portion of citrate was metabolized through the TCA cycle to oxaloacetate, while another fraction was converted to isocitrate and subsequently entered the glyoxylate shunt, generating succinate and glyoxylate. These intermediates were further converted to (S)-malate via malate synthase and subsequently oxidized to oxaloacetate by malate dehydrogenase. Activation of the glyoxylate pathway is considered an important strategy for microorganisms to maintain carbon efficiency and energy balance under high organic acid conditions. In this context, Mr, which retains high metabolic activity under low pH conditions, may play a critical role in sustaining TCA cycle and glyoxylate shunt fluxes during fermentation [33,44].

Overall, after 6 days of multi-microbial fermentation, several carbohydrate-related metabolites were significantly downregulated, while multiple organic acids, including pyruvate, citrate, and malate, showed pronounced decreases. These results indicated that the microbial consortium facilitated coordinated organic acid consumption through interconnected pathways involving glycolysis, the pentose phosphate pathway, the TCA cycle, and the glyoxylate shunt. Collectively, functional differentiation and metabolic complementarity among the three microorganisms effectively redirected carbon flux from carbohydrate degradation toward organic acid metabolism, forming a metabolic basis for efficient deacidification of citrus pulp.

#### 3.5.2. Biosynthesis of Amino Acids and Their Derivatives

After 6 days of multi-microbial fermentation, amino acid biosynthesis emerged as one of the most significantly altered metabolic categories in citrus pulp. Metabolomic analysis revealed that glycolysis and the tricarboxylic acid (TCA) cycle functioned as central metabolic hubs, tightly coupled with multiple amino acid metabolic pathways, indicating coordinated redistribution of carbon and nitrogen fluxes during fermentation. Such metabolic reprogramming is characteristic of multi-microbial systems adapting to acidic substrates and achieving efficient substrate utilization [45].

During fermentation, the glycolytic intermediate phosphoenolpyruvate (PEP) was redirected toward the formation of phenylpyruvate and tyrosine precursors, providing carbon skeletons for aromatic amino acid biosynthesis. This process was likely associated with the metabolic activity of Hp, which has been reported to possess active glycolytic flux and aromatic amino acid precursor metabolism [46]. Meanwhile, pyruvate, the terminal product of glycolysis, served as a key precursor for amino acid biosynthesis, being converted into 2-oxoisovalerate, alanine, and 2-oxobutanoate. Among these, 2-oxoisovalerate is a central intermediate for valine and leucine biosynthesis, whereas 2-oxobutanoate contributes to the synthesis of threonine, glycine, serine, and cysteine.

Bs likely played a dominant role in these amino acid metabolic pathways. As a metabolically versatile bacterium, *B. subtilis* possesses complete biosynthetic routes for branched-chain and several non-essential amino acids and efficiently utilized pyruvate and TCA cycle intermediates as amino acid precursors [47,48]. In addition, pyruvate-derived acetyl-CoA entered the TCA cycle, supplying carbon backbones for leucine and lysine biosynthesis, highlighting the central role of Bs in linking carbohydrate metabolism, organic acid utilization, and amino acid synthesis.

Within the TCA cycle, 2-oxoglutarate served as a key precursor for glutamate synthesis, which in turn acted as a metabolic node for arginine, ornithine, and citrulline metabolism. This branch was likely co-regulated by Bs and Mr. Previous studies have shown that *Monascus* sp. maintain active nitrogen metabolism under low-pH conditions and participate in glutamate-related transformations [49,50]. Moreover, glutamate could be decarboxylated to γ-aminobutyric acid (GABA), which was subsequently converted into succinate via the GABA shunt and re-entered the TCA cycle. This pathway provides an alternative route linking amino acid metabolism with energy generation and may contribute to acid stress buffering and metabolic stability during fermentation [15,51].

Collectively, these results indicated that, during the multi-microbial fermentation, organic acids were not only consumed through glycolysis, the TCA cycle, and the glyoxylate pathway, but were also redistributed into amino acid biosynthesis through tightly coupled carbon–nitrogen metabolism. Simultaneously, certain amino acid-derived intermediates flowed back into the TCA cycle as succinate or fumarate, forming an integrated metabolic loop of organic acid consumption, amino acid synthesis, and energy rebalancing. This coordinated metabolic architecture provided a mechanistic explanation for the high efficiency and stability of organic acid reduction observed in the multi-microbial fermentation of citrus pulp.

### 3.6. Metabolic Reprogramming Towards High-Value Bioactive Compounds and Evaluation of Biostimulant Potential

Beyond the degradation of organic acids, the multi-microbial consortium facilitated a profound metabolic reprogramming. The carbon and nitrogen fluxes were redirected to synthesize high-value bioactive compounds, primarily functional lipids and plant hormones. This section elucidates the biosynthesis of these compounds and validates their practical application in improving the growth and nutritional quality of edible sprouts.

#### 3.6.1. Lipid Profile Remodeling and Biosynthesis of Functional Lipids

Concurrently with organic acid depletion, a significant upregulation of lipid biosynthesis pathways was observed (Figure 6), indicating a metabolic flux shift where acetyl-CoA derived from acid catabolism was channeled into de novo fatty acid biosynthesis for carbon conservation [52]. This metabolic reprogramming led to the specific accumulation of high-value bioactive lipids. Notably, nervonic acid, a critical component for myelin biosynthesis, increased by 30-fold. This accumulation is likely driven by the robust fatty acid elongation systems of *Monascus* spp., which efficiently utilize the expanded acetyl-CoA pool for long-chain fatty acid assembly [53]. Similarly, 10-hydroxy-2-decenoic acid (10-HDA), a bioactive compound typically exclusive to royal jelly, was identified [54]. Its presence suggests the activation of fungal ω-hydroxylation pathways, where medium-chain fatty acids are enzymatically modified—a phenomenon observed in diverse microbial biotransformations under carbon-rich conditions [55]. Furthermore, the detection of 2-hydroxybutyric acid and uric acid correlates with accelerated L-threonine decomposition and intensified nucleic acid turnover (Figure 7), respectively, reflecting the high metabolic vitality and nitrogen-adaptation strategies of the multi-microbial consortium [56,57]. Beyond improving nutritional value, these unsaturated fatty acids can function as exogenous elicitors to trigger plant secondary metabolism [54,58]. Consequently, this lipid remodeling provides the chemical basis for the biostimulant effects validated in the subsequent sprout cultivation (Section 3.6.2).

#### 3.6.2. IAA Biosynthesis and Application in Edible Sprout Production

**Figure 8:**
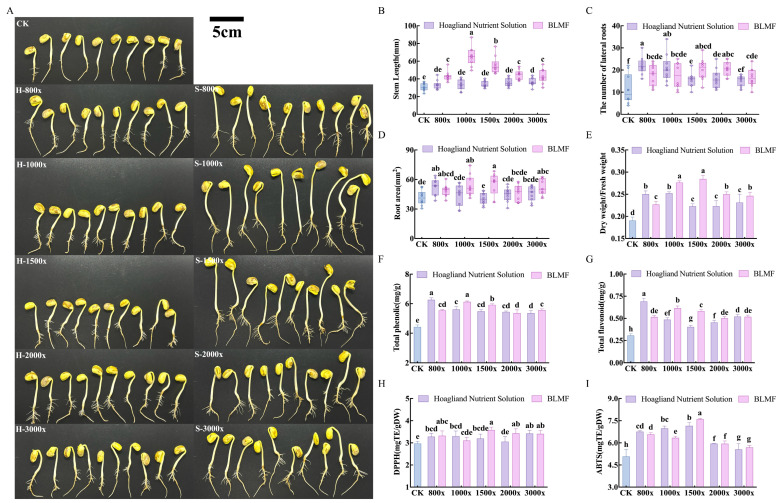
Evaluation of the biostimulant potential of BsHpMrF fermentation products on the growth performance and nutritional quality of soybean sprouts. (**A**) Representative phenotypes of sprouts cultivated under different treatments: sterile water (CK), Hoagland nutrient solution (H), and BsHpMrF fermentation products (S) at various dilution ratios (800×–3000×). (**B**) Stem length. (**C**) Number of lateral roots. (**D**) Root area. (**E**) Ratio of dry weight to fresh weight. (**F**) Total phenolic. (**G**) Total flavonoid. (**H**) DPPH radical scavenging activity. (**I**) ABTS radical scavenging activity. Note: multi-microbial fermentation (BsHpMrF). Different letters (a, b, c, etc.) within the same group point indicate significant differences at *p* < 0.05 according to Duncan’s multiple range test.

Consistent with the upregulation of tryptophan metabolism, the fermentation products accumulated 414.28 ± 0.21 mg/L of IAA, a concentration that aligns with highly efficient plant growth-promoting rhizobacteria [59]. Interestingly, while standard inorganic nutrient solutions like Hoagland solution primarily support basic vegetative growth through mineral supplementation, the fermentation products exhibited a superior capacity for morphological remodeling. Upon application at optimal dilutions (1000×–1500×), sprout morphogenesis was significantly enhanced: stem length increased by 116.08% ± 0.48, and lateral root number surged 2.03-fold to 17.60 ± 0.51 (Figure 8A–C).

This robust root architecture exceeds the effects typically observed with purely mineral-based fertilization, suggesting that the fermentation products efficacy stems from more than just nutrient availability. The “auxin-driven” phenotype is likely a result of the high IAA titer working in tandem with other fermentation-derived metabolites, such as bioactive lipids and organic acid derivatives, which may act as co-factors for hormone perception [60].

Furthermore, the fermentation products functioned as a potent metabolic elicitor—a feature largely absents in conventional nutrient solutions—boosting total phenolic and flavonoid contents to peaks of 6.13 ± 0.06 mg/g and 0.62 ± 0.02 mg/g, respectively. This secondary metabolic surge was particularly evident at the optimal dilution of 1500×, where the BsHpMrF-treated group exhibited significantly elevated DPPH (3.58 ± 0.13 mg TE/g DW) and ABTS (7.60 ± 0.06 mg TE/g DW) scavenging activities compared to the control groups (*p* < 0.05, Figure 8H,I). These results suggest that appropriately diluted fermentation products can effectively enhance the antioxidant capacity of the plants. Unlike Hoagland solution, which focuses on biomass accumulation, these results confirm the fermented pulp as a dual-action biostimulant. It not only drives vegetative growth but also proactively triggers the plant’s defense-related pathways, thereby fortifying nutritional quality [58].

## 4. Conclusions

In this study, a functional consortium (BsHpMrF) comprising *B. subtilis* L4 (Bs), *H. pseudoguilliermondii* B4 (Hp), and *M. ruber* 10910 (Mr) was constructed to achieve efficient deacidification and high-value transformation of citrus pulp. The synergistic cooperation not only accelerated organic acid mineralization via the activation of the TCA cycle and glyoxylate shunt but also enhanced acid tolerance, characterized by membrane lipid remodeling (specifically increasing unsaturation levels). Moreover, the fermentation enriched the matrix with bioactive lipids and IAA, acting as a potent biostimulant that remodeled soybean root architecture and enhanced antioxidant accumulation. However, given that these metabolic shifts were inferred primarily from untargeted metabolomics, future studies employing multi-omics or genetic approaches are needed to pinpoint specific regulatory pathways. Furthermore, rigorous safety assessments for potential mycotoxins (e.g., citrinin) and pilot-scale trials remain essential to validate the biosafety and economic feasibility of this valorization strategy within the circular food economy.

## Data Availability

The original contributions presented in this study are included in the article/Supplementary Materials. Further inquiries can be directed to the corresponding authors.

## Supplementary Materials

The following supporting information can be downloaded at: https://www.mdpi.com/article/10.3390/foods15081276/s1, Figure S1: pH-based screening of co-fermentation partners for *Hanseniaspora pseudoguilliermondii* B4; Table S1: Factors and levels of orthogonal test; Table S2: Results of the L9 (3^4^) orthogonal experimental design and range analysis for the optimization of deacidification conditions; Figure S2: Relative abundances of metabolites in the fermentation supernatants of the unfermented group (F0) and citrus pulp fermented for 6 days (F6, 144 h); Table S3: Quantitative profiles and relative changes of significantly regulated metabolites between unfermented citrus pulp (F0) and citrus pulp fermented for 6 days (F6). Table S4: Relative quantification results of significantly up-regulated and down-regulated metabolites (VIP > 1.0, *p* < 0.05) identified in Figure 7.
