# Supplementary material for: Biological Deacidification and High-Value Transformation of Acidic Citrus Pulp by Multi-Microbial Fermentation
Source: Foods. 2026 Apr 8;15(8):1276. doi: 10.3390/foods15081276 (PMC13115456; doi:10.3390/foods15081276)
Supplement: Supplementary file 1 [file foods-15-01276-s001.zip › foods-4208234-supplementary.pdf]

Table S1 Factors and levels of orthogonal test

Levels	Factors		
	A Inoculum size/%	B Temperature/℃	C Time/h
1	5	28	96
2	10	30	120
3	15	32	144

Table S2 Results of the L9 (3^4) orthogonal experimental design and range analysis for the optimization of deacidification conditions

Number	Factor			Result
	Inoculum size /%	Temperature/°C	Time/h	Total acid/ mmol/mL NaOH
1	-1(5)	-1(28)	-1(96)	33.25
2	-1(5)	0(30)	+1(144)	21.00
3	-1(5)	+1(32)	0(120)	23.50
4	0(10)	-1(28)	0(120)	24.00
5	0(10)	0(30)	-1(96)	25.25
6	0(10)	+1(32)	+1(144)	16.75
7	+1(15)	-1(28)	+1(144)	20.50
8	+1(15)	0(30)	0(120)	21.25
9	+1(15)	+1(32)	-1(96)	28.00
K1	77.75	77.75	86.50	
K2	66.00	67.50	68.75	
K3	69.75	68.25	58.25	
k1	25.92	25.92	28.83	
k2	22.00	22.50	22.92	
k3	23.25	22.75	19.42	
R	3.92	3.42	9.42	

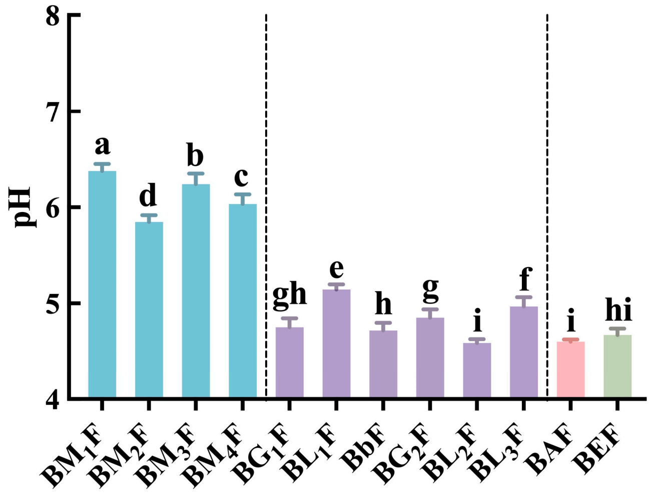

Figure S1. pH-based screening of co-fermentation partners for *Hanseniaspora pseudoguilliermondii* B4. The pH values of citrus pulp after 5-day fermentation by B4 coupled with various strains: BM1F-BM4F, *Monascus* spp. (10910, 3.702, GIM 3.592, and MS127); BG1F-BL3F, *Bacillus* spp. (GZ3-e16, L4, bf4-88, GZ3-L38, L20, and L16); BAF, *Aspergillus niger*; BEF, *Eurotium cristatum*. Dashed lines separate microbial genera. Data represent mean ± SD (n=3). Different letters (a, b, c, etc.) within the same time point indicate significant differences at $p < 0.05$ according to Duncan's multiple range test.

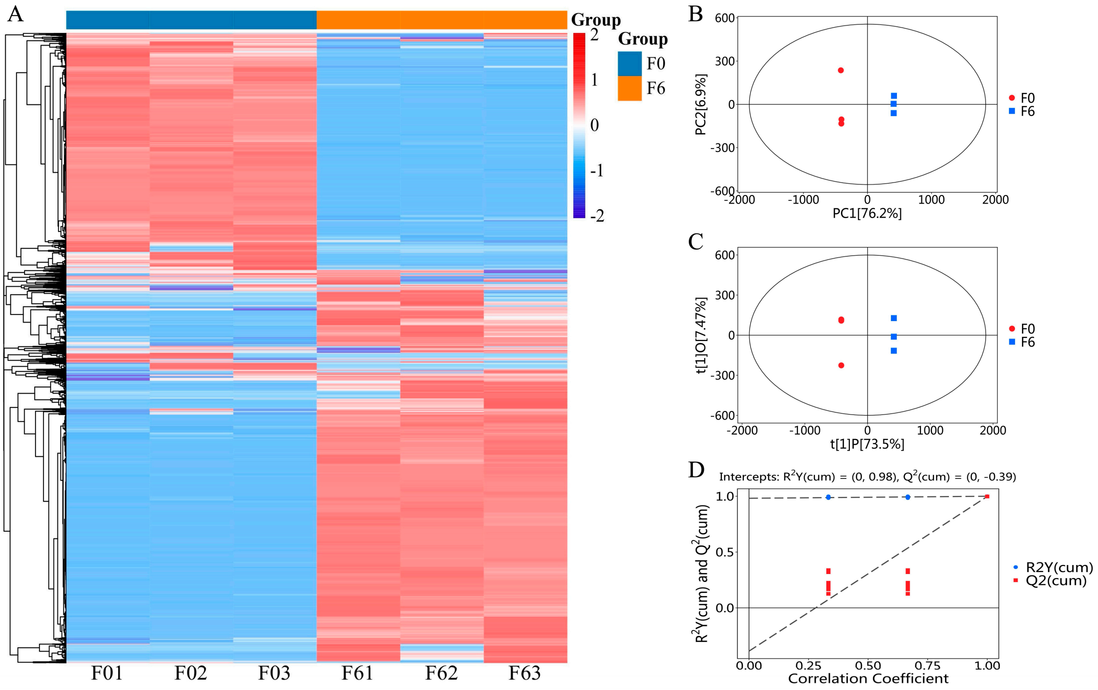

Figure S2. Relative abundances of metabolites in the fermentation supernatants of the unfermented group (F0) and citrus pulp fermented for 6 days (F6, 144h). (A) Heatmap of differential metabolites; labels F01–F03 and F61–F63 represent three independent biological replicates for the F0 and F6 groups, respectively. Red and blue colors indicate increased and decreased metabolite levels (VIP > 1, $p < 0.05$). (B) PCA score plot. (C) OPLS-DA score plot comparing F0 and F6. (D) Permutation test of the OPLS-DA model for F0 and F6.

Table S3 Quantitative profiles and relative changes of significantly regulated metabolites between unfermented citrus pulp (F0) and citrus pulp fermented for 6 days (F6).

Metabolite	Class	VIP	*p*-value	LogFC	Type	Relative changes
3,4-Dihydroxybenzoic acid (Protocatechuic acid)	Benzenoids	1.09	0.00	1.51	up	284.51%
Pentadecanoic acid	Lipids and lipid-like molecules	1.17	0.00	4.43	up	2151.85%
cis-9-Palmitoleic acid	Lipids and lipid-like molecules	1.15	0.02	2.03	up	407.57%
Myristic acid	Lipids and lipid-like molecules	1.15	0.02	2.30	up	492.04%
Royal jelly acid	Organic acids and derivatives	1.17	0.00	1.89	up	370.74%
Propionic acid	Organic acids and derivatives	1.16	0.00	1.40	up	263.36%
Glucose 6-phosphate	Organic oxygen compounds	1.13	0.00	0.99	up	198.15%
2-Hydroxypalmitic acid	Lipids and lipid-like molecules	1.16	0.00	2.20	up	458.99%
9-Oxooctadecanoic acid	Lipids and lipid-like molecules	1.16	0.01	5.02	up	3235.35%
Nervonic acid	Lipids and lipid-like molecules	1.16	0.01	4.96	up	3118.27%
Citric acid	Organic acids and derivatives	1.16	0.00	-6.13	down	1.43%
Uric acid	Organoheterocyclic compounds	1.16	0.00	-6.01	down	2.07%
Glyceric acid	Organic oxygen compounds	1.17	0.00	-5.60	down	4.67%
Poncirin	Phenylpropanoids and polyketides	1.11	0.00	-4.42	down	0.02%
Deoxyguanosine	Nucleosides, nucleotides, and analogues	1.16	0.00	-12.25	down	4.29%
Malic acid	Organic acids and derivatives	1.16	0.00	-4.54	down	0.55%
alpha-Ketoglutaric acid (alpha-KG)	Organic acids and derivatives	1.16	0.01	-7.50	down	4.99%
Pyroglutamic acid	Organic acids and derivatives	1.16	0.00	-4.33	down	0.80%
Leucylphenylalanine	Organic acids and derivatives	1.17	0.00	-6.96	down	1.50%
L-Valyl-L-phenylalanine	Organic acids and derivatives	1.17	0.00	-6.06	down	2.69%

Relative changes (F6/F0, %): This value was calculated as the ratio of the mean peak intensity (relative quantification) of each metabolite on Day 6 (F6) to that on Day 0 (F0), expressed as a percentage.

Table S4 Relative quantification results of significantly up-regulated and down-regulated metabolites (VIP > 1.0, *p*<0.05) identified in Figure 7.

Metabolite	Mean F0	Mean F6	LogFC	Type
a-D-Glucose-1P	6.24543E-07	4.20925E-05	2.475776375	up
2-Oxoisocaproate	3.5786E-05	0.001070145	4.902266472	up
2-Oxoisovalerate	2.46245E-05	0.000348654	3.823627207	up
Valine	9.05454E-06	5.82691E-05	2.686017471	up
HPP	6.8055E-07	1.47017E-05	4.433135421	up
Glycine	2.56777E-06	4.22928E-05	4.041825777	up
S-Acetyl-dihydrolipoamide-E	2.89978E-06	1.35345E-05	2.222623125	up
Homocitrate	7.2088E-07	9.33822E-06	3.695315776	up
Glutamine	2.09525E-06	8.99567E-06	2.102107149	up
Citrulline	2.29312E-05	5.86415E-05	1.354609888	up
a-D-Glucose-6P	4.65662E-06	2.35001E-06	-0.98661885	down
Pyruvate	0.000605106	8.3415E-05	-2.858809906	down
D-Glucono-1,5- lactone	7.15065E-05	1.15878E-05	-2.625462957	down
2-Dehvdro-3-deoxv-D-gluconate	0.005657099	2.5808E-06	-11.09802545	down
Asparagine	0.00038131	2.62174E-06	-7.184296414	down
Aspartate	4.35438E-05	1.24577E-06	-5.127359606	down
Alanine	4.40287E-05	3.62525E-05	-0.280363393	down
L-Arginino-succinate	1.24982E-05	3.72348E-06	-1.746991016	down
Serine	5.3524E-05	2.18361E-06	-4.6153988	down
O-Acetyl-serine	0.000108625	4.77179E-06	-4.5086818	down
Tyrosine	1.71416E-05	5.60737E-06	-1.612108552	down
Phenylpyruvate	2.16836E-05	1.42209E-05	-0.608596027	down
Homoserine	1.87547E-05	3.0413E-06	-2.624497302	down
Isoleucine	7.67748E-05	6.35636E-05	-0.272432294	down
(S)-Malate	9.31706E-06	5.42815E-07	-4.101343319	down
Isocitrate	0.02363069	0.000336952	-6.131974612	down
Succinate	0.000445767	0.000311851	-0.515430214	down

Glutamate	0.000240018	8.46802E-06	-4.824973063	down
N-Acetyl-omithine	2.07601E-05	1.32469E-06	-3.970091787	down
2-Oxoadipate	1.01823E-05	2.74666E-07	-5.212239123	down
Lysine	5.56525E-06	2.00234E-06	-1.47475523	down
Citrate	0.02363069	0.000336952	-6.131974612	down
Isocitrate	0.02363069	0.000336952	-6.131974612	down
2-Oxoglutarate	3.68202E-05	4.21022E-07	-6.4504562	down
Succinate	0.000445767	0.000311851	-0.515430214	down
4-Aminobutanoate	0.000416983	6.35767E-06	-6.035346814	down
